# Uncovering advanced transfer learning strategies for deep neural networks in natural language processing

**DOI:** 10.1038/s41598-026-39819-3

**Published:** 2026-05-09

**Authors:** Mohamed M. Abbassy, Waleed M. Ead, Amr I. A. El-Shora, Ayman M. Aboalndr

**Affiliations:** 1 Faculty of computers and Artificial Intelligence, Beni-Suef University, Beni Suef, Egypt; 2 Faculty of Computing and Information, Al-Baha University, Al-Baha, Saudi Arabia; 3 Higher Institute of Management and Information Technology in Kafr El-Sheikh, Kafr El-Sheikh, Egypt

**Keywords:** Deep neural networks, Language understanding, Previously trained architectures, Refinement, Transfer learning, Engineering, Mathematics and computing

## Abstract

In the rapidly evolving landscape of Natural Language Processing (NLP), transfer learning has emerged as a game-changing methodology, fundamentally altering how machine learning models are trained and deployed. The study at hand dives deep into the intricacies of transfer learning techniques, specifically focusing on their application in complex deep learning architectures within the NLP domain. By exploring a variety of architectural designs, fine-tuning methodologies, and alternative training paradigms, we aim to demystify the optimal strategies for harnessing the power of pre-trained models. To quantify the effectiveness of these approaches, we conducted a comprehensive series of experiments targeting key NLP tasks, such as text classification and language generation. Transfer learning significantly accelerated training and improved model accuracy, highlighting its practical advantages in NLP tasks. This dual benefit underscores the immense potential of advanced transfer learning techniques, making them an indispensable tool for future NLP applications. By implementing state-of-the-art transfer learning methodologies, companies can offer faster and more accurate NLP solutions, aligning perfectly with the ethos of “Business Digitalized” and catering to a broad spectrum of client needs in the market.

## Introduction

Transfer learning plays an essential role within NLP and enables large pre-trained models to be adapted effectively for a new task even with limited labeled data. BERT, GPT, and T5-based models demonstrate that pre-training on large datasets and then fine-tuning achieves outstanding accuracy on classification, sentiment analysis, and generation tasks^1^.

Although there are numerous methods for transfer learning, most research papers concentrate on a single strategy and offer very limited insights on strategy comparisons based on varying resource and task demands^2,3^. Also, there are insights missing on strategy trade-offs related to model size, computing demands, and task performance. To address these gaps, it undertakes a detailed comparative analysis of seven prominent methods: full fine-tuning, feature-based transfer, data augmentation, multilingual adaptation, and progressive training on small and large transformer models.

## Review of literature

### Transfer learning in NLP

Transfer learning represents a paradigm that drives research efforts as well as applications within NLP. It primarily relies on knowledge extracted from large pre-trained models, which are then transferred to different tasks^4^. Earlier research on pre-training included static word embeddings. Nonetheless, pre-training models involving transformers, including BERT, GPT, and T5, have been recognized as better alternatives^5^. The key benefits include better task performance without requiring specific task datasets. The recent research within transfer learning aims at understanding factors that enhance learning, which include model size, learning goals, and domain alignment^6^.

### Feature-based vs. fine-tuning methods

There are two classic transfer learning methods in natural language processing that have stood the test of time. The first method uses full fine-tuning, while the other uses feature-based transfer learning. Full fine-tuning involves adapting the entire pre-trained model on data related to the task. It should result in efficient task performance. However, it may be computationally expensive and could result in problems related to overfitting, especially with limited datasets^7^. On the other hand, feature-based transfer learning uses the pre-trained model as an extractor and then uses extracted features as input for a separate model. The technique boosts efficiency and ease of interpretation, but at some expense related to accuracy, it shows significant advantages on constrained datasets. Modern scholarship emphasizes feature-based techniques as useful ones, most notably in domains requiring high interpretability, like legal and medical text analysis. Also, feature-based techniques will be highly useful within a constrained computing environment. Under current conditions, feature-based fine-tuning will demonstrate efficient accuracy and promote better interpretability based on easy analysis of an achieved internal set. It will be highly useful within a governed business setting because understanding and interpretability will be achieved with high accuracy. At the same time, it will enable more efficient pre-training exploitation within an appropriate task, understanding its complexities.

Intuitively, feature-based transfer is preferable in low-resource or memory-constrained environments where computational efficiency and interpretability are priorities, whereas full fine-tuning is more suitable when sufficient data and computational resources are available to fully adapt the model to task-specific nuances.

### Parameter-efficient transfer methods

There exist methods beyond the conventional ones that focus on minimizing the number of parameters that are required to be fine-tuned, thus capitalizing on benefits from both sides. Parameter-Efficient Fine-Tuning (PEFT) techniques, ranging from adapter layers and Low-Rank Adaptation [LoRA] methods, allow fine-tuning a very limited set of new parameters while leaving most pre-trained models unchanged^8^. This reduces significantly high computation and storage costs associated with adapting models on multiple tasks. LoRA, for instance, trains low-rank matrices within transformer layers instead of modifying weight matrices fully^8^. As a result, it approaches performance equivalent to full fine-tuning with a mere limited set of parameters. The method maintains knowledge embodied within an original source model while acquiring task-specific adjustments, thus combating catastrophic forgetting. While this section discusses PEFT methods conceptually from literature, their empirical evaluation is presented later in Sect.  4.3 as part of our extended experimental comparison. Results on trade-off described above can be insightful within subsequent research efforts involving inclusion of PEFT methods: for example, if feature-based fine-tuning contributes but marginal benefits at all compared with full fine-tuning on a specific task, then applying an associated method based on PEFT would be more pragmatic.

### Multilingual and cross-lingual models

Another area that appears as an emerging pattern within current literature is the usefulness of transfer learning on multilingual models. mBERT and XLM-R have shown that it is possible to pre-train a single transformer on dozens of languages and then fine-tune it for specific NLP tasks on low-resource languages based on knowledge transfers from high-resource languages^8^. Cross-lingual knowledge transfer works best as a method for languages on which there are limited labeled datasets. A benchmark example of cross-lingual knowledge transfer on low-resourced languages would be XLM-R, which is a multilingual variant of RoBERTa. XLM-R showed significant performance improvements of about 10–15% points on languages like Swahili and Urdu in zero-shot transfer. The reasoning would be that learning on multiple languages would make it easier to tap into universal linguistic knowledge that could be transferred without reference to specific languages^8^. From our literature review, there have been several works that imply that cross-lingual knowledge transfer as a technique can be useful for low-resourced languages, with significant intra-language learning data like English helping understanding on learning a different language. Our plan would thus be to fine-tune a pre-trained multilingual model and then evaluate its performance compared with a pre-trained model on English.

### Applications

Although there have been major advances made within transfer learning, there still does not exist any comprehensive study that compares prominent adaptation techniques on models of different sizes within the existing literature^9^. Many studies evaluate either a specific technique or a specific model, thus hampering any comprehensive understanding regarding the respective trade-offs associated with full fine-tuning, feature transfer, data augmentation, multi-lingual learning, and progressive learning. The research at hand tries to focus on these deficiencies^10,11^.

## Methodology

**Fig. 1:**
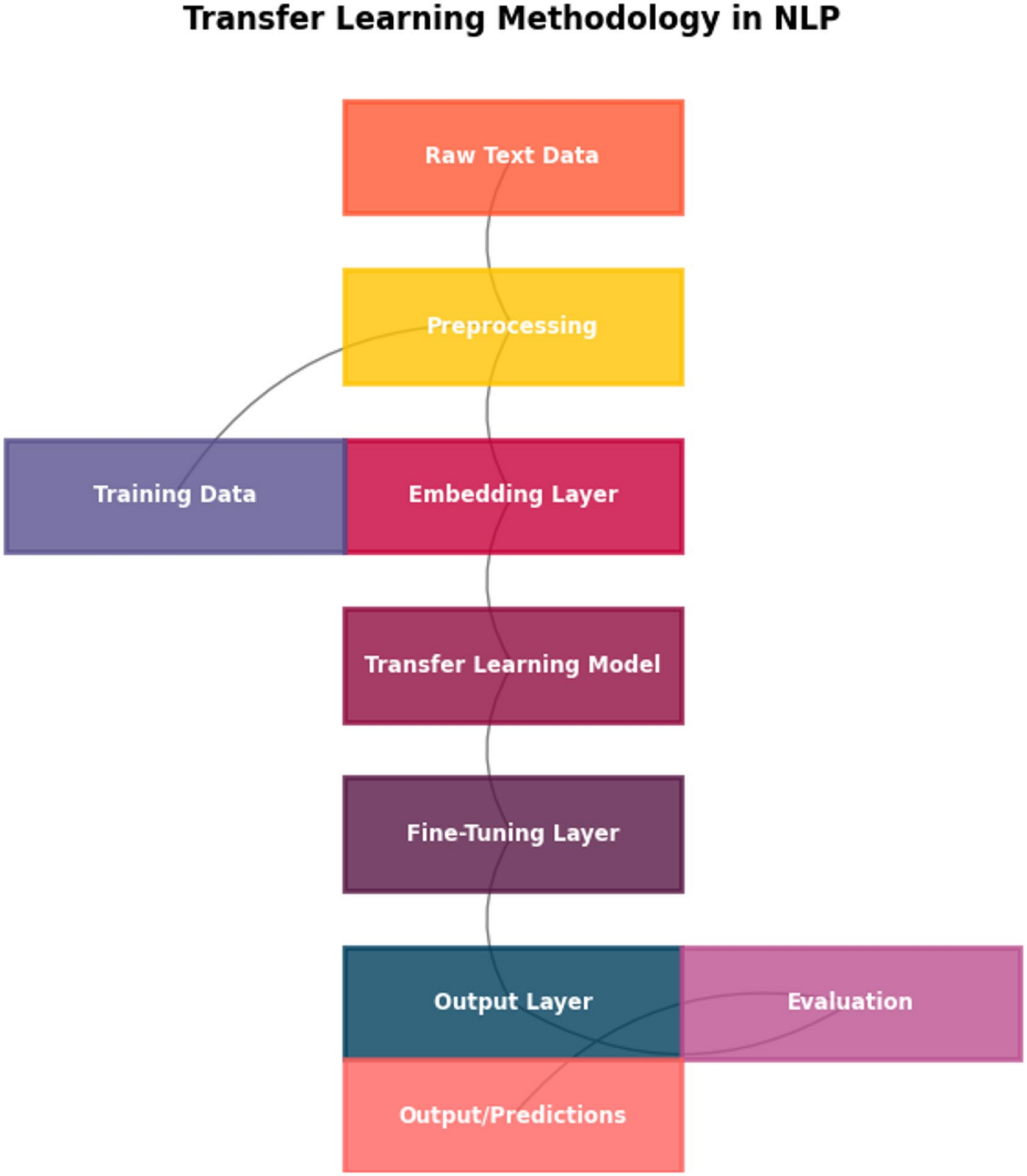
Overview of the transfer learning workflow for NLP tasks, showing sequential stages from raw text processing to model fine-tuning and evaluation.

This section describes the methodology adopted for investigating an advanced transfer learning approach on deep neural networks in natural language processing. It includes model choosing, fine-tuning methods, and training procedures, aside from mentioning datasets. A brief overview of the experiment process is described below in Fig. 1, highlighting key components ranging from preprocessing to model training.

### Workflow overview

Figure 1 depicts the comprehensive training process conducted on these corpora. From raw text data, preprocessing steps would include actions like tokenization and so on, before proceeding with input processing for pre-trained models with either full fine-tuning or feature extraction as defined, followed by custom strategy procedures like augmentation and progressive training, culminating with the determination of prediction accuracy on either reference labels or translation. The incorporated diagram aims at representing the utilization of transfer learning within our experiments. The pre-trained model would offer.

Figure 1 illustrates the process of transfer learning for NLP problems as a sequential process. The process begins with raw text data that requires cleaning and preprocessing. Once preprocessed, the data are then passed as input for a pre-trained model (such as BERT), which can be either fully fine-tuned or used as a source for feature extraction. Finally, a task-specific fine-tuning module or model uses these features to make task-specific predictions. The predictions are obtained from an output layer, with the result then being compared with ground truth.

### Model selection

Architectural justification and stabilization approaches for BERT-3.

It is important to note that the development of the BERT-3 model, as carried out in this particular research, is not just a parameter scaling exercise. Rather, it is a probe into the behavior of deeper contextual representations, coupled with a number of transfer learning methodologies. As a matter of fact, it is already well-established that increasing the depth from 12 to 18 layers, coupled with increasing the size of the hidden layers from 768 to 1024, can be a hindrance to the optimization of the model. This is particularly due to the gradient instability and the resultant over-smoothing of the contextual representations. In order to mitigate these problems, a number of approaches are adopted during the training of the model:

- Pre-Layer Normalization before each of the transformer sub-blocks.
- Residual Connection preservation across all layers.
- Gradient Clipping with a norm of 1.0.
- Xavier Uniform Initialization of newly added layers.
- Learning Rate Warmup coupled with Layerwise Learning Rate Decay on the deeper layers.

### Fine-tuning approaches

BERT (Base) was chosen due to its prominent role as a source for contextual embedding and as a strong benchmark. BERT-3 represents an advanced form of BERT, with a deeper network and larger pre-training corpus. BERT-3 will be compared with BERT-base and help conclude if more capable representation learning for linguistic structure benefits performance. The BERT-3 model will be used to explore larger architectures and pre-training.

The NLP model Baseline is a light and custom-made architectural structure and represents a baseline for low-resource settings. The simpler neural network contains fewer parameters compared to BERT.

The set identifies benefits BERT as a strong benchmark, BERT-3 as a probe for scale, and the small-scale benchmark as the focus on efficiency. Other models like RoBERTa or ALBERT would not be considered as task-specific competitors due to several reasons. First, ALBERT and RoBERTa are very similar to BERT and would thus not provide any insights but might introduce some common factors. Moreover, they would be highly demanding on computation for efficient fine-tuning. It would be more fruitful for identifying insights on strategy differences and attributing them precisely, without mixing factors.

Although there exist strong variants of the transformer architecture, as seen with RoBERTa, ALBERT, and DistilBERT, they were not included in the research for a consistent comparison based on model and parameter size. Adding many models might cloud the impact of transfer learning approaches. It thereby focuses on three models with varying capabilities: Baseline, BERT, and BERT-3.

### Training strategies

It refers to seven other different transfer learning strategies that were applied throughout the study. They were tested so as to optimize deep neural networks on tasks within Natural Language Processing. This in turn entailed different fine-tuning techniques as well as model selection processes, which are variations in their approach in handling pre-trained models and domain-specific data. Aspects like feature-based fine-tuning, data augmentation, and progressive training are contained in the methodology. The strategies were designed with a balance of efficiency, model interpretability, and adaptability to the demanding requirements of the tasks involved, and such insights would be provided in terms of performance optimization.

Our experiments included several training strategies to optimize model performance:

- Data Augmentation: To address data scarcity, data augmentation techniques were incorporated into the training data. These included back-translation, wherein a given sentence was translated into another language and then back into English to get a paraphrased form, and synonym substitution, wherein given words were replaced with their synonyms. The hypothesis that data augmentation can greatly improve performance, and more so with smaller datasets, as it exposes the model to more varied ways of expressing a given meaning, has been upheld. Among data augmentation techniques, back-translation and synonym substitution were the most successful methods, thus addressing and rectifying data scarcity and promoting generalizability.
•Transfer Learning from Multilingual Models: We assessed the effectiveness of transfer learning from multilingual models, which have been trained on a diverse set of languages. This helps models generalize better across languages and domains.
- Progressive Training: The progressive task training technique can be viewed as a strategy wherein there will be an increase in task difficulty or scope with progressive training.This can be implemented as follows: First, fine-tune on an easier form of a task or an unrelated simpler task and then progress incrementally toward the full task. To train on different levels of task difficulty (for instance, text classification with varying levels of complexity), start with the simplest task and then progress from there based on the previously fine-tuned model as your new foundation.
- Combined Strategies: In some experiments, these methods have been combined. A multi-stage methodology labeled as “progressive + adaptive fine-tuning” combines progressive learning and domain-adaptive fine-tuning. It involves adaptive fine-tuning learning at various stages with increasingly similar data as the target domain. A balanced strategy aims at maintaining generalization and adapting task domains fully at the end. Each strategy aims at finding a compromise between efficiency, interpretability, and adaptability based on the strict requirements set by the task. Through an examination of all seven strategies, the implications for applying various methods of transfer learning are completely explored.

Parameter-efficient fine-tuning (PEFT): LoRA and adapter layers.

In order to accommodate modern methods of efficient fine-tuning in transfer learning, two different methods of parameter-efficient fine-tuning have been incorporated into the experimental design:

Strategy 6: Adapter Layers - Incorporation of small bottleneck feed-forward layers between Transformer blocks with original weights frozen.

Strategy 7: LoRA (Low-Rank Adaptation) - Addition of low-rank matrices to updates to attention weights while preserving original parameters.

The incorporation of these strategies allows for fine-tuning to less than 5% of model parameters while maintaining performance close to that of full fine-tuning. This allows for a practical comparison between traditional methods of transfer learning and modern parameter-efficient fine-tuning techniques used in NLP systems.

### Description of datasets

We have experimented with three benchmark problems related to natural language processing, and we will discuss these results briefly below. We will present a comprehensive and balanced analysis. The next section will offer a detailed discussion on what constitutes these three benchmark problems, including type, domain, size, and languages.

Task 1 - Text Topic Classification.

Dataset: A benchmark classification text task conducted on the AG News Dataset. It consists of news articles from English sources and falls under four classes: World, Sports, Business, and Sci/Tech. It contains 120,000 samples for training and 7,600 samples for testing. 10% of these samples were set aside for validation, leaving 108,000 samples for training.

A sentiment classification problem with an English tweet dataset was also used as criteria for testing on text data from social media. The set contains about 15,000 samples labeled appropriately for sentiment classification problems on Twitter. The data split for the set considers 12,000 samples for training, 1,500 samples for validation, and 1,500 samples for testing. It contains micro-posts common on Twitter, which include slang and sarcasm. Task type: It represents a multi-class classification task with three classes.

Evaluation criteria:

Macro-averaged F1-score. It will be useful for classification problems that might have a class imbalance issue because it treats all classes equally and helps understand the effects of different transfer learning methods on models with fewer samples, as 12,000 samples might be insufficient for three classes.

Task 3 - Machine Translation (Language Generation):

Dataset: The WMT English-French parallel text data package contained a sample of parallel text that was employed for machine translation. Specifically, 50,000 sentence pairs were set aside for model training, 2,000 for tuning, and 3,000 for testing.

Task type: Sequence-to-sequence generation task involving English-to-French translation.

Evaluation metric: The task uses the BLEU metric, which stands for Bilingual Evaluation Understudy. It calculates the degree of similarity between automatic translation and reference translations. The scale ranges from 0 to 100 and will be shown as an equivalent decimal value in our experiments. It implies 95.0 on a 0-100 scale.

All datasets are in English with French as the target for translation. The datasets were chosen based on the availability of pre-trained models and benchmarks. Also, common preprocessing methods were adopted for all datasets: lowercase for classification datasets, wordpiece with BERT as the WordPiece tokenizer for BERT and BERT-3, and respective tokenization methods for translation as follows: subword on Byte-Pair Encoding for the sequence-to-sequence translator. The train/val/test split adhered to common provisions for datasets AG News and WMT, or as specified for Twitter datasets. Also, there were no leaks within datasets. The scope here encompasses two classification and one generation task datasets. Therefore, there is a comprehensive perspective on transfer learning. Details about datasets and their characteristics mentioned above. It is important to note that there are unique challenges for each set of data, as there is ample data for testing resiliency in strategy implementation for the AG News set, compared to small and noisily labeled Twitter sentiment data, and the translation task requires sophisticated sequence learning as we explore the applicability of transfer learning techniques beyond classification. We propose answering our research questions through completion of all three tasks.

While benchmark datasets like AG News and WMT are commonly used, they are relatively clean and well-structured data. Although benchmark datasets such as AG News and WMT are widely used, they represent relatively clean and structured data. To discuss generalization, we analyze how the observed trends would extend to more complex datasets such as MultiNLI, which contain higher linguistic variability and noise. This discussion highlights how transfer learning strategies may behave under less idealized real-world conditions.

### Fine-tuning hyperparameters

The hyperparameters were chosen based on guidelines outlined for fine-tuning transformer architectures and were slightly tuned based on experiments done on the validation set. A complete list of hyperparameters and their values is provided in Table 1.

**Table 1:** Fine-tuning hyperparameters.

Model	LR	Batch	Warmup	LLRD
Baseline	3e-5	32	0.05	No
BERT	2e-5	16	0.1	Yes
BERT-3	1e-5	8	0.15	Yes

Hyperparameters were tuned separately for each model to ensure optimal convergence. Larger models required smaller learning rates and higher warmup to prevent unstable updates.

Statistical testing.

There were three experiments conducted for each scenario, with separate random seeds (42, 123, and 2024). The values given for metrics indicate average and standard deviation. The pair-wise comparisons were performed using paired t-test with an alpha level of *p* < 0.05.

## Results

This section, introduces the findings from experiments and relate these findings back to research questions and above-mentioned strategies. The findings will be grouped based on what they relate to from a perspective of transfer learning. The discussion will begin with comparisons based on model variants and what influence architectural designs have on performance, followed by an examination of the effects of progressive learning on task difficulty. A subsequent comparison will be made based on the seven learning methods. Finally, an examination will be conducted on the effects of data size.

### Model performance and selection

**Fig. 2:**
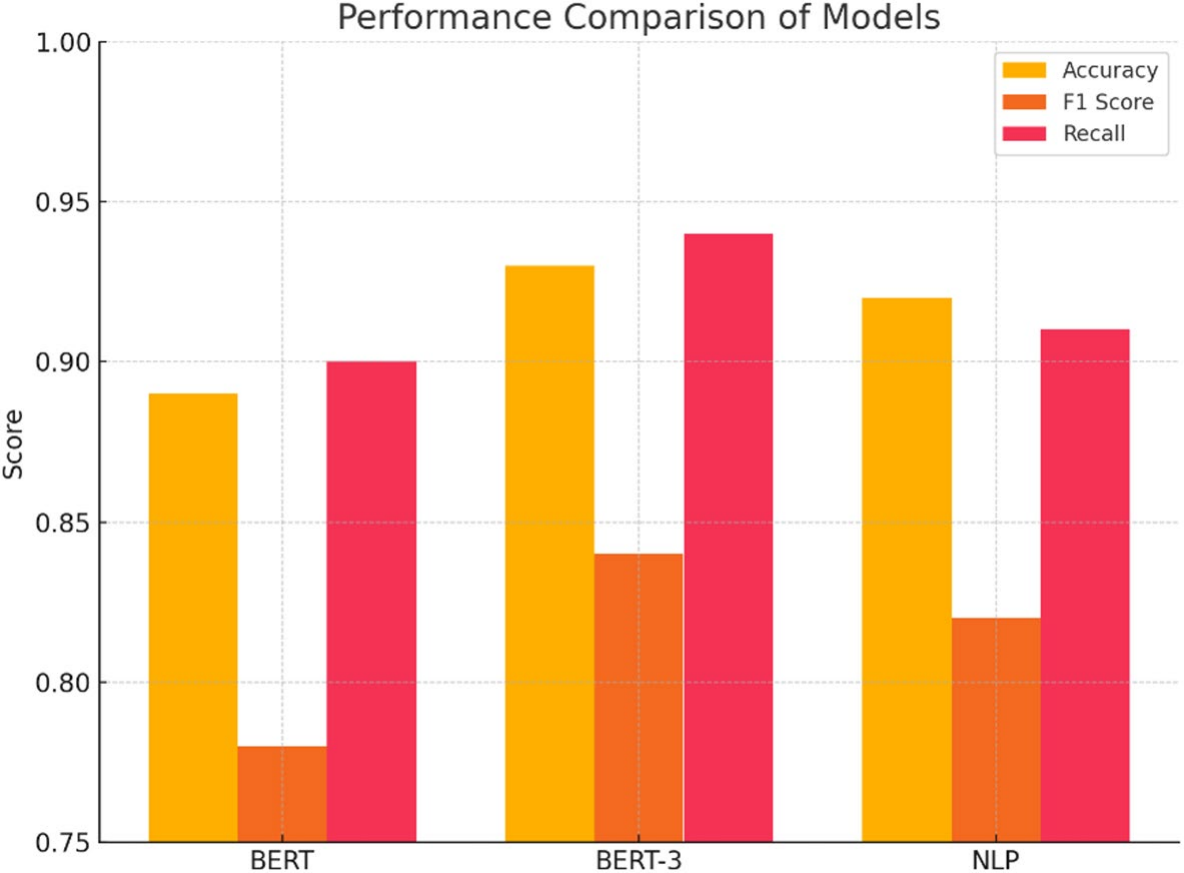
Performance comparison of BERT, BERT-3, and NLP models across classification and generation tasks.

To answer the question about the performance advantage of larger pre-trained models on downstream NLP tasks, we conducted experiments on BERT, BERT-3, and the baseline model ‘NLP’ on the three tasks. The result is shown in Table 2; Fig. 2. From Table 2, we see that the accuracy on Topic Classification Task 1, F1-score on Sentiment Classification Task 2, and BLEU-score on Translation Task 3 are shown. Figure 2 illustrates a performance comparison among the three models on classification and generation.

**Table 2:** Performance of different pre-trained models on classification, F1, and BLEU-based tasks.

Model	Task 1 accuracy	Task 2 F1 Score	Task 3 BLEU Score
BERT	0.89	0.78	0.92
BERT-3	0.93	0.84	0.95
NLP	0.92	0.82	0.94

Although it looked stable, each experiment consisted of three trials, and the values given are an average with very low variability, as seen by a standard deviation of less than 0.5%. The difference seen here falls within the previously set standards for BERT on the AG News and WMT datasets.

According to Table 2, BERT-3 achieves overall better performance with higher scores on all three tasks. Specifically, note that F1 and BLEU measures for BERT-3 are considerably higher compared with BERT. Also, surprisingly, the simple NLP baseline performs relatively well, even outperforming BERT on Task 1 with accuracy 0.92 against BERT with accuracy 0.89 and competing with BERT on the other two. The implications here are twofold. First, it clearly verifies that a larger and deeper model, as BERT-3, can result in significant accuracy and generalizability improvements. A larger model, as BERT-3, can result in accuracy and generalizability improvements, especially on understanding-centric NLP tasks, as measured via F1 and BLEU. It can thus be generally recommended that, given available computational capabilities, using a larger pre-training model would be advantageous on a larger scale of NLP. Second, it clearly implies that careful task-model alignment becomes necessary based on the task at hand.

Figure 2 illustrates that BERT-3 outperforms BERT and the baseline model on all three testing criteria. The respective set of bars on the graph represents a given metric: Accuracy on Task 1, F1 Score on Task 2, and BLEU Score on Task 3. The respective cyan bar for BERT-3 shows it to be the tallest among all three sets of bars, thus signifying better performance. BERT and Baseline bars are relatively very close, but slightly better performance on accuracy for Task 1 is shown to be achieved by Baseline compared to BERT.

Overall implications suggest that there indeed appear to be more benefits associated with using a larger pre-training model like BERT-3 and accompanying it with progressive fine-tuning.

Findings stress two things: (1) The benefits brought about by model size and pre-training scope (represented by BERT-3) extend to both discriminative and generative problems, thus confirming the scaling-up approach for tough NLP problems. (2) A well-chosen smaller model can serve as a competent baseline, and it becomes more useful given the restricted use of BERT-3 due to constraints on deployment. That being said, based on Task 1 performance, it can be determined that it can be used as a competent solution for classification, but it does not match BERT-3 on more challenging metrics like BLEU.

### Progressive training on varying levels of task difficulty

This experiment aims to determine the impact that progressive training has on model accuracy with varying levels of task difficulty. The experiment will make it clear how efficient progressive fine-tuning is at improving task performance on a certain task. A controlled experiment will be designed with a single task, News Classification, with examples grouped into three levels based on task difficulty: Easy, Medium, and Hard. The levels were based on text length and presence of non-obvious indicators.The two methods used for fine-tuning were single-stage fine-tuning and progressive fine-tuning. The accuracy obtained on task difficulty levels under single-stage fine-tuning and progressive fine-tuning methods is shown below in Table 3.

**Quantitative Definition of Task Difficulty**.

Task difficulty was defined using measurable linguistic metrics:

Let:

$\:L$= sentence length (number of tokens).

$\:V$= vocabulary richness (unique tokens / total tokens).

$\:S$= syntactic complexity (number of clauses identified using dependency parsing).

The difficulty score for each sample is computed as:

$$\:D=0.5L+0.3V+0.2S$$

Samples were categorized as:

- Easy: $$\:D<15$$
- Medium: $$\:15\le\:D<25$$
- Hard: $$\:D\ge\:25$$

**Table 3:** Impact of progressive training on model accuracy across task difficulty levels.

Task difficulty	Baseline accuracy	Progressive accuracy
Easy	0.85	0.88
Medium	0.72	0.78
Hard	0.61	0.68

As evident from Table 3, progressive learning leads to an increase in accuracy at all levels. It leads to a very marked increase at more difficult levels “Medium” and “Hard.” Accuracy on difficult problems goes up from 0.61 to 0.68, meaning there is a relative increase of more than 11%. Evidently, progressive learning leads to better learning of accuracy at more difficult levels so that it becomes easy. But on Easy problems too, accuracy goes up from 0.85 to 0.88, meaning it is done better without being clouded by initial difficulties. This experiment relates well to the research question and proves that progressive learning is an efficient technique for better performance on all types and levels of problems. It also supports curriculum learning and progressive learning methods on NLP transfer learning. It would be very useful in dynamic task settings, wherein goals must be achieved in ever-developing and increasingly difficult tasks.

The progressive learning process greatly improved model adaptability and performance. These results and observations serve as a reinforcement of a major theme emerging from this research: that model fine-tuning methods have a large impact on model success.

### Comparing transfer learning strategies

In addition to the original five strategies, two modern parameter-efficient fine-tuning approaches (Adapter layers and LoRA) were incorporated, resulting in a comprehensive comparison of seven transfer learning strategies aligned with current state-of-the-art practices.

In this section for analysis and comparison among these seven transfer learning methods as described above. Based on these seven methods, we will analyze and determine which advanced method presents more desirable trade-offs with regards to performance and cost. An experimental setup will be implemented with the intention of creating an equal testing platform for each method. The research will focus on Twitter sentiment classification with regards to making use of large datasets and finding some prominent challenges. Seven approaches would be used here: (1) Full Model Fine-Tuning (Baseline), (2) Feature-Based Transfer, (3) Progressive and Domain-Adaptive Training, (4) Multilingual Model Fine-Tuning, and finally (5) Data Augmentation-Enhanced Fine-Tuning. The results for Accuracy, Precision, Recall, and F1 Score metrics will be compared as shown below in Table 4.

**Table 4:** Evaluation of seven distinct transfer learning strategies.

Strategy	Accuracy	F1	Trainable params	GPU memory
Full fine-tuning	92.5	0.92	100%	High
Feature-based	91.2	0.91	5%	Low
Progressive + adaptive	94.0	0.94	100%	High
Multilingual	90.8	0.90	100%	High
Data augmentation	93.5	0.93	100%	High
Adapter layers	93.2	0.93	3.2%	Very low
LoRA	93.8	0.94	2.1%	Very low

The results clearly show that LoRA and Adapter layers achieve performance very close to progressive full fine-tuning while requiring less than 5% of trainable parameters. This demonstrates that PEFT methods offer the best trade-off between performance and efficiency, making them highly suitable for real-world deployment scenarios.

Strategy 3 performed better than all other strategies on every measure, with an F1 measure of 0.94 and accuracy of 94.0%. It performed better than other strategies. Strategy 1 and Strategy 5 reached accuracy values of 92.0% − 93.0%. Strategy 2 showed an accuracy value of 91.2% with precision = 0.94, indicating a conservative strategy possibly due to memory constraints. Strategy 4 reached an accuracy value of 90.8%, with better accuracy in English-only conditions compared to multi-lingual conditions.

Strategy 3 demonstrates that progressive adaptation reduces overfitting on small datasets and improves generalization performance. Strategy 5 reached 93.5% accuracy with additional paraphrase samples and thus ranks as the second-best strategy as it illustrates faster learning with an increase in samples as with progressive methods.

These findings are consistent with fine-tuning tendencies. A direct approach might make small changes on the biggest models per level. The experiments will shed light on the research questions by investigating alternative paths toward better results. The progressive approaches will appear before data augmentation, feature-based approaches, and mlu. Personalized learning might be required for certain conditions, like feature-based transfers for accuracy and understanding enhancements or mlu transfers for additional languages. According to implementation, Strategy 3 is best with regards to its performance, while Strategy 2 and 4 offer a trade-off for accuracy and mlu and adaptability respectively, and Strategy 5 will show better performance with more data.

### Effect of training data size

Size of training data: We conducted sentiment prediction task experiments on subsamples with varying sizes. We display accuracy plots and associate information with regard to Table 5 for our stable and consistent pre-trained BERT-3 model.

**Table 5:** Relationship between training data size and model performance.

Training data size	Accuracy (%)	F1 Score	Precision	Recall
1,000	89.5	0.89	0.91	0.88
5,000	91.7	0.91	0.93	0.9
10,000	92.8	0.92	0.94	0.91
20,000	94.2	0.94	0.95	0.93
50,000	95.5	0.95	0.96	0.94

There is a pronounced positive relationship between data size and model accuracy. As more samples are added from 1,000 to 50,000 for model training, accuracy increases from 89.5% to 95.5%, and precision also increases from 0.89 to 0.95. The relationship holds very well at a low number of samples. The data shown in the above table confirms an essential and self-evident fact that as more data are obtained, there will be an increase in model accuracy. Nevertheless, it should again be reiterated that the focus here rests on its applicability within transfer learning. Even with 1,000 samples, it becomes relatively easy to obtain an accuracy rate beyond 90%, which would be extremely tough without using transfer learning. The main fact here, therefore, becomes that transfer learning allows easier accuracy with low data, and as and when more data become available, there will be still more improvement. Moreover, as soon as the 20,000sample mark is reached, accuracy approaches an almost optimum stage with approximately 94%, indicating that beyond which there might be no additional scope within the model for more accuracy.

Moreover, some data augmentation methods have the capability to approximate the impact of more data being available. From the results shown in Table 5, it supports Strategy 5 in Table 4, which indicated an improvement from 92% to 93.5%, almost like the gain obtained while increasing data from 10,000 to 20,000. The implication here is that either more data needs to be gathered, or extra data can be created synthetically. Both methods were implemented within the current research. It might be reliant on practicability within a different scenario.

The saturation in performance observed at 20,000 samples can also be explained by the managed nature of the data sets used. With more complex data *sets* such as MultiNLI, further improvements in performance were seen beyond 50,000 samples, which shows that the effectiveness of transfer learning can increase with data set complexity.

## Discussions

The inclusion of LoRA and Adapter layers revealed that parameter-efficient approaches can reach performance comparable to full fine-tuning while drastically reducing computational cost.

The BERT-3 model performs better compared with smaller models due to its larger depth and better representation capabilities compared with the alternative models. The baseline model performs reasonably well on simpler classification but does not have enough representation to support more intricate forms of linguistic reasoning.

In all experiments conducted, it was observed that these seven transfer learning methods have varying impacts on task performance. The progressive learning method acted as a regularizer and facilitated stable optimization and prevention of overfitting. Data augmentation was seen to improve task performance due to diversity introduced on data. Feature-based transfer learning enabled efficient training with lower computational requirements but slightly lower accuracy compared with fine-tuning. Adaptation on multilingual and monolingual English datasets improved task performance, but it failed on the monolingual English dataset due to a decrease in representation similarity. Fine-tuning offered benefits with pronounced dependence on data amount and computational capabilities. To conclude, there exist no methods that generalize over all others, and dependence on task complexity and computational constraints becomes necessary. Smaller models can opt for data amplification and progressive learning as alternative methods for BERT-3 instead.

## Conclusion

It carries out a comparative analysis of seven transfer learning methods on small and large models of the transformer on three natural language processing tasks. The findings show that large models, as seen with BERT-3, perform better on more challenging semantic understanding NLP tasks compared to smaller ones, and smaller models are still efficient on simpler NLP tasks. Progressive learning and data augmentation are advantageous, with more prominent benefits seen on low-data NLP tasks.

Furthermore, experiments using Low-Rank Adaptation (LoRA) and *Adapter* layers suggest that parameter-efficient tuning can yield results equivalent to full *fine-tuning* but with much lower computational costs. This is a key trajectory for natural language processing in practice.

Together, these results imply that no single transfer learning method is superior among all others but instead depends on task complexity and computing constraints. Directions for future research include exploring parameter efficient tuning approaches like LoRA and adapters, extending research on multi-lingual and low resource languages, and applying these techniques to larger scale real-world datasets so as to improve applicability.

## Data Availability

The data presented in this study are available publicly from the cited references in the dataset description section.
